# Effects of Hematological Parameters and Plasma Components of Starry Flounder, *Platichthys stellatus,* by Waterborne Copper Exposure

**DOI:** 10.3390/ani15111549

**Published:** 2025-05-25

**Authors:** Su-Min An, Cheol Young Choi, Jun-Hwan Kim

**Affiliations:** 1 Department of Aquatic Life and Medical Science, Sun Moon University, Asan 31460, Republic of Korea; mjcha7474@naver.com; 2 Division of Marine BioScience, National Korea Maritime and Ocean University, Busan 49112, Republic of Korea; 3 Department of Aquatic Life Medicine, Jeju National University, Jeju 63243, Republic of Korea; 4 Department of Marine Life Science, Jeju National University, Jeju 63243, Republic of Korea

**Keywords:** copper exposure, LC_50_, hematological parameters, plasma components, starry flounder

## Abstract

Starry flounder (*Platichthys stellatus*) (weight 96.42 ± 19.17 g, length 20.65 ± 1.04 cm) were exposed to waterborne copper at 0, 0.5, 1, 3, 6, 12, 24, and 48 mg Cu^2+^/L for 96 h. The lethal concentration 50 (LC_50_) of the *P. stellatus* exposed to waterborne copper was 15.644 mg Cu^2+^/L. Hemoglobin, hematocrit, and RBC count were significantly decreased by waterborne copper exposure. MCV (mean corpuscular volume) (µL) and MCHC (mean corpuscular hemoglobin concentration) (%) were also significantly decreased. The inorganic components, plasma calcium, and plasma magnesium were significantly increased. The organic components, such as plasma glucose, were significantly increased. In enzymatic components, the AST and ALT were also significantly increased by copper exposure. The results of this study indicate that exposure to copper may have effects on the survival rates and hematological parameters of the *P. stellatus*.

## Simple Summary

Copper is indiscriminately released into the natural environment through both natural processes and anthropogenic activities, posing a potential toxic threat to aquatic organisms. This study investigated the physiological effects of acute copper exposure over 96 h in starry flounder (*Platichthys stellatus*). The 96 h median lethal concentration (LC_50_) of copper in starry flounder exposed to high concentrations was determined to be 15.644 mg Cu^2+^/L, indicating the tolerance threshold to copper toxicity. Hematological parameters, including hemoglobin (Hb), hematocrit (Ht), and red blood cell (RBC) count, were significantly decreased. In contrast, plasma inorganic components (Ca, Mg), organic components (glucose), and enzymatic components (AST, ALT) were significantly increased. These findings suggest that the physiological responses of *P*. *stellatus* following acute copper exposure can serve as reliable standard indicators for assessing the tolerance limits of fish to copper toxicity.

## 1. Introduction

Copper (Cu) is an essential trace element that plays an important role in various biological functions such as growth, development, and reproduction in fish [1,2]. Copper can directly affect aquatic life when it is indiscriminately discharged into the natural environment as a pollutant due to natural and anthropogenic activities (natural resources, pesticides, electronic device disinfectants, etc.) [3]. In particular, it is used as an effective algae remover and disinfectant to remove aquatic organism pathogens in aquaculture environments [4]. Copper can occur naturally in aquatic environments at low concentrations (typically 2–30 µg/L), but it can be released into water at high levels due to industrial wastewater from the aquaculture, agriculture, and mining industries, reaching concentrations of 50–560 µg/L, which are above the toxic level for aquatic organisms. High levels of copper exposure can be fatal to aquatic organisms [5,6,7].

Copper is absorbed into the body of fish primarily through the gills but also via dietary intake as it accumulates in living organisms [8]. Since the gills are the main organs that continuously come into contact with the external environment and absorb toxic substances, including metals, they can act as target organs to identify the toxic effects in fish. Recent studies have also reported that heavy metals tend to bioaccumulate in specific tissues, such as the gills and muscles, further supporting their role as key indicators for environmental toxicity assessment [9]. Copper toxicity can cause ion regulation disorders in the gills, which promotes the loss of Na^+^ and Cl^−^ through the inhibition of Na^+^/K^+^-ATPase activity, and it induces increased gill permeability and osmotic pressure disorders [10]. Copper in the water can be absorbed into the body through fish gill epithelial cells and can cause major accumulation in liver tissue, which can affect the enzymatic metabolism of liver tissue and cause increased lipoperoxidation (LPO) and serious histological changes in liver tissue due to the promotion of oxidative stress [10,11]. Copper can accumulate in various tissues at high exposure concentrations, which can cause various physiological and histological changes in fish, including anorexia, reduced growth, ion loss, and structural changes, which can lead to mass mortality [12].

In exposure experiments, the lethal concentration (LC_50_) is the concentration at which 50% of organisms die due to a toxic substance, which is used as an important indicator to evaluate the toxic effects and risks of toxic substances on fish through the potential effects on the survival of experimental fish [13,14]. The toxic effects of acute exposure to metal substances vary depending on many factors, such as exposure concentration, exposure period, and age and size of the organism; in particular, fish can easily metabolize and accumulate metals such as copper in their bodies, so they can serve as representative organisms for monitoring aquatic ecosystems [5]. Copper is an essential trace element for aquatic organisms at low concentrations (2–30 µg/L), but exposure to high concentrations (50–560 µg/L) can cause serious toxicity and lead to mass mortality [15]. Lethal concentration (LC_50_) is an indicator of the sensitivity of fish when exposed to toxic substances; the higher the LC_50_, the higher the tolerance limit of fish to toxic substances [16,17]. Therefore, the LC_50_, according to acute exposure to waterborne copper, will provide a standard for the tolerance limit of experimental fish and establish a standard for the safe concentration according to the presence of copper in water.

Hematological parameters are used as an important indicator to evaluate the health status of various fish after metal exposure, such as waterborne copper, which can be different depending on the type of toxic substance [18,19]. Copper in water is rapidly accumulated in the circulatory system through absorption through the gills, resulting in various physiological responses, including blood balance disorders, and it can cause hematological disorders by inducing tissue damage in the hematopoietic organs [19]. In addition, waterborne copper exposure can cause respiratory and ion regulatory dysfunction due to structural changes in the gill epithelium and increased ion permeability, which can lead to increases in hematological characteristics such as hematocrit, red blood cell count, and hemoglobin concentration. Recent studies have demonstrated that such physiological changes, often associated with heavy metal bioaccumulation in fish tissues, can be effectively evaluated using advanced techniques such as flow cytometry [20]. Therefore, the hematological characteristics and plasma components of fish after acute exposure to waterborne copper will provide reference indicators for evaluating the toxic effects of blood on fish after exposure to copper toxicity.

Fish are used as a representative model for evaluating the toxicity of pollutants because their biochemical and physiological responses are similar to those of mammals [21]. Starry flounder (*Platichthys stellatus*) belongs to the *Platichthys* family of the flatfish family (Pleuronectidae), which is widely distributed across the northern Pacific Ocean, including the coasts of Korea, Japan, China, Russia (Sea of Okhotsk and Bering Sea), Alaska, and as far south as northern California in the United States [22]. It is an euryhaline species and can also be found in brackish waters or downstream of rivers. Starry flounder is actively being cultured in Korea for its high marketability due to its excellent taste and high nutritional value (source of protein and fatty acids). Production increases every year, reaching approximately 3373 tons in 2018 and 3669 tons in 2019 [23]. However, little research has been conducted on the reference indicator according to acute exposure to waterborne copper. Therefore, the purpose of this study was to evaluate the acute toxic effects of waterborne copper exposure in *P*. *stellatus* by determining the 96 h LC_50_ value and assessing hematological and plasma biochemical responses at sublethal concentrations. The results aim to provide baseline data for understanding copper-induced physiological disturbances and to contribute to the establishment of safe environmental thresholds for this species.

## 2. Materials and Methods

### 2.1. Experimental Fish and Experimental Environment

The *P. stellatus* (weight 96.42 ± 19.17 g, total length 20.65 ± 1.04 cm) used in this experiment was supplied from a fish farm in Jeju Island and was used after being reared and acclimated in the laboratory. The *P. stellatus* was raised in a circular tank before the experiment, and the reared individuals were used in this experiment. A 30 L square experimental tank was used, and exposure was performed for 96 h at specific concentration levels (0, 0.5, 1, 3, 12, 24, and 48 mg Cu^2+^/L). The experiment was conducted with a total of 42 animals (7 copper concentration groups x 6 animals per experimental group) for exposure, and water quality measurements (water temperature, dissolved oxygen, salinity, and pH) were measured using a portable water quality analyzer (YSI-Professional plus, YSI Inc., Yellow Springs, OH, USA). The dissolved oxygen (DO) level was maintained at 6.17 ± 0.14 mg/L, and water temperature, salinity, and pH were kept within optimal ranges to minimize environmental stress on the fish. In this study, copper exposure was performed using copper chloride (CuCl_2_) to make a standard stock solution of 10,000 mg Cu^2+^/L, and exposure was performed at an appropriate concentration for each tank.

### 2.2. Lethal Concentration (LC_50_)

To determine the lethal concentration due to waterborne copper exposure, the presence of death in each tank was checked at 0, 1, 3, 6, 12, 24, 48, 72, and 96 h after copper exposure, and dead individuals were immediately removed. Specifically, after 96 h of copper exposure, all fish (n = 6) in the control (0 mg/L), 0.5 mg/L, and 1 mg/L groups survived (n = 6), whereas partial mortality was observed in the 3 mg/L (n = 5), 12 mg/L (n = 4), 24 mg/L (n = 1), and 48 mg/L (n = 0) groups. Based on the final death individuals due to waterborne copper exposure after 96 h, the lethal concentration value was calculated using a statistical program (SPSS Inc., Chicago, IL, USA, probit model).

### 2.3. Hematological Characteristics

Hematological parameters were analyzed by blood collection using surviving individuals after 96 h of exposure to waterborne copper. Blood was collected using a heparinized syringe (Sigma Chemical, St. Louis, MO, USA), and hemoglobin and hematocrit were measured immediately after blood collection. Hemoglobin levels were measured using the cyano-methemoglobin method using a clinical kit (Asan Pharm. Co., Ltd., Seoul, Republic of Korea). Hematocrit was measured using a micro-hematocrit reader after centrifuging blood into a capillary tube at 12,000 rpm for 10 min in a micro-hematocrit centrifuge (hanil, Gimpo, Republic of Korea). RBC counts were counted under an optical microscope using a hemocytometer (Marienfeld Superior, Lauda-Konigshofen, Germany) after diluting blood 400-fold with Hendrick’s diluting solution. Based on the results of hemoglobin, hematocrit, and RBC count, mean corpuscular volume (MCV), mean corpuscular hemoglobin (MCH), and mean corpuscular hemoglobin concentration (MCHC) were calculated using the following methods:

MCV (µL) = Hematocrit (%)/RBC count (10^7^/µL) × 10

MCH (pg) = Hemoglobin (g/dL)/RBC count (10^7^/µL) × 10

MCHC (%) = Hemoglobin (g/dL)/Hematocrit (%) × 100

### 2.4. Plasma Components

To analyze changes in plasma components due to acute exposure to copper, blood was collected and centrifuged at 3000× *g* at 4 °C for 15 min to separate plasma. Calcium and magnesium were measured as plasma inorganic components. Calcium was measured according to the OCPC method, and magnesium was measured according to the Xylidyl blue-I method using a clinical test kit (Asan Pharm. Co., Ltd.). Glucose, cholesterol, and total protein were analyzed as plasma organic components. Plasma glucose was measured according to the GOD/POD method, and cholesterol and total protein were measured according to the colorimetric method and the Biuret method using commercially available clinical test kits (Asan Pharm. Co., Ltd.). Aspartate aminotransferase (AST), alanine aminotransminase (ALT), and alkaline phosphatase (ALP) were analyzed as plasma enzyme activities. AST and ALT were analyzed according to the Reitman–Frankel method at 505 nm, and ALP was analyzed according to the King–King method at 500 nm using a clinical test kit (Asan Pharm. Co., Ltd.). Although procedural blanks were not separately analyzed, the clinical test kits and analytical instruments used in this study are regularly calibrated and maintained according to the manufacturers’ quality control guidelines. All experimental procedures, including blood collection and sample handling, were conducted in strict compliance with institutional and international safety protocols to minimize contamination and ensure the accuracy and reliability of the analytical results.

### 2.5. Statistical Analysis Method

This experimental analysis used the surviving fish from each of the seven copper exposure concentrations (0 to 48 mg/L). Initially, six fish were used per group; however, the number of fish analyzed varied depending on survival after 96 h of exposure. The statistical significance of the experimental analysis results was determined by conducting an ANOVA test using the SPSS/PC+ statistical package software version 20 (SPSS Inc., Chicago, IL, USA), and Tukey’s multiple range test was used to determine significance when *p* < 0.05.

### 2.6. Ethics Approval and Consent to Participate

This study was conducted with the research ethics approval of the Institutional Animal Care and Use Committee of the Jeju University (2025-0016). In addition, all researchers have completed animal protection, animal welfare, and animal experimentation conducted by the Institutional Animal Care and Use Committee of Jeju University.

## 3. Results

### 3.1. Survival Rate and Lethal Concentration (LC_50_)

**Figure 1:**
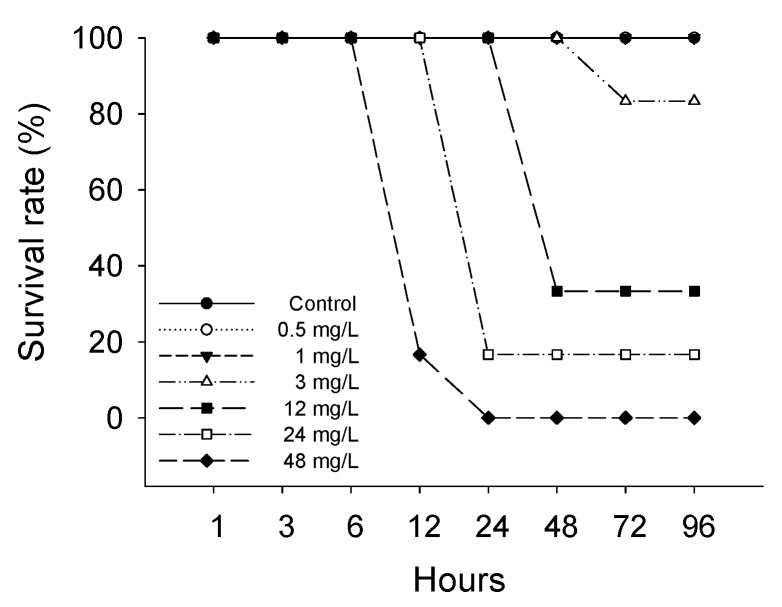
Survival rate of starry flounder, *Platichthys stellatus*, exposed to waterborne copper for 96 h.

The survival rate of *P. stellatus* gradually decreased with increasing concentrations of waterborne copper exposure (Figure 1). In the control group and at 0.5, 1 mg Cu^2+^/L, all individuals survived throughout the 96 h exposure period. Mortality first appeared at 72 h in the 3 mg Cu^2+^/L group, with 16.67% mortality by 96 h. Higher concentrations induced earlier and greater mortality, reaching 100% at 48 mg Cu^2+^/L after 96 h. The lethal concentration 50 (LC_50_) of the *P. stellatus* exposed to waterborne copper is shown in Table 1. The lethal concentration 50 of the *P. stellatus* exposed to waterborne copper was 15.644 mg Cu^2+^/L.

### 3.2. Hematological Parameters

**Figure 2:**
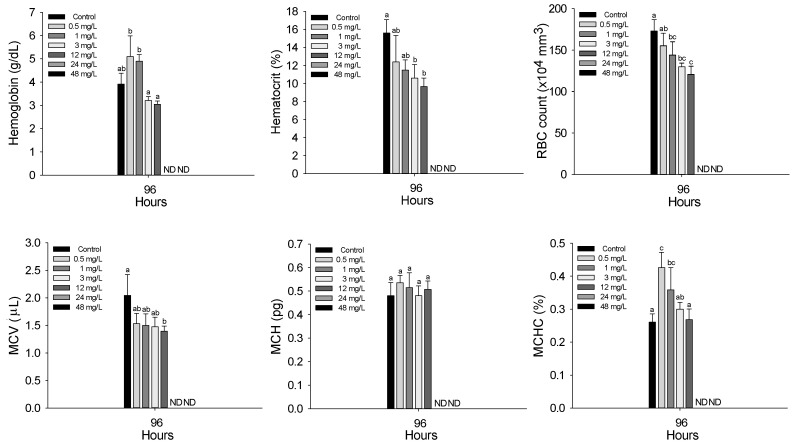
Hematological parameters of starry flounder, *Platichthys stellatus*, exposed to waterborne copper for 96 h. Values with different letters indicate that they are significantly different (*p* < 0.05) after one-way ANOVA following Tukey’s multiple range test. Values are expressed as mean ± standard deviation (SD). ND: no data due to mortality.

The hematological characteristics of *P. stellatus* after acute exposure to waterborne copper are shown in Figure 2. All hematological and plasma parameters were analyzed only in surviving fish; thus, physiological changes were observed only at concentrations ≤ 12 mg Cu^2+^/L, as no fish survived at 24 and 48 mg Cu^2+^/L. After acute copper exposure, hemoglobin concentration significantly decreased at concentrations of 0.5 mg Cu^2+^/L or higher (*p* < 0.05). Hematocrit showed a significant decrease at 3 mg Cu^2+^/L and above, while RBC count decreased significantly at 1 mg Cu^2+^/L or higher (*p* < 0.05). Among the calculated erythrocyte indices, MCV significantly decreased only at 12 mg Cu^2+^/L, MCH showed no significant changes, and MCHC significantly decreased at concentrations of 0.5 mg Cu^2+^/L or higher (*p* < 0.05).

### 3.3. Plasma Components

**Figure 3:**
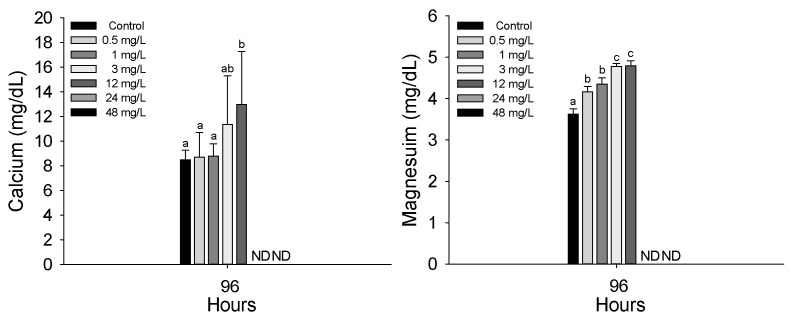
Inorganic plasma components of starry flounder, *Platichthys stellatus*, exposed to waterborne copper for 96 h. Values with different letters indicate that they are significantly different (*p* < 0.05) after one-way ANOVA following Tukey’s multiple range test. Values are expressed as mean ± standard deviation (SD). ND: no data due to mortality.

**Figure 4:**
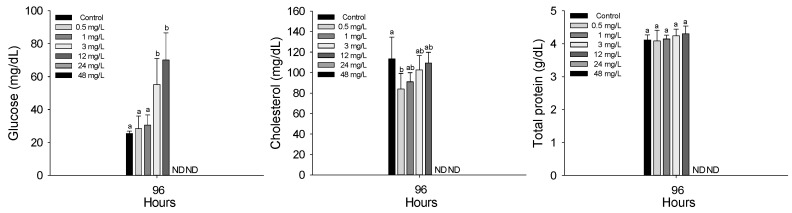
Organic plasma components of starry flounder, *Platichthys stellatus*, exposed to waterborne copper for 96 h. Values with different letters indicate that they are significantly different (*p* < 0.05) after one-way ANOVA following Tukey’s multiple range test. Values are expressed as mean ± standard deviation (SD). ND: no data due to mortality.

**Figure 5:**
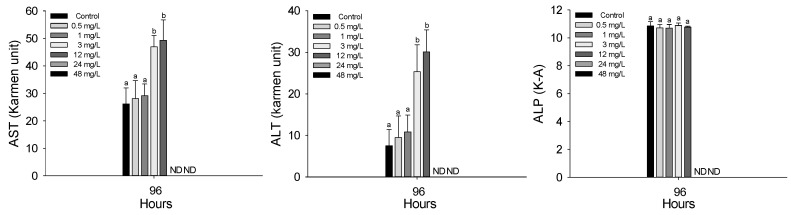
Enzymatic plasma components of starry flounder, *Platichthys stellatus*, exposed to waterborne copper for 96 h. Values with different letters indicate that they are significantly different (*p* < 0.05) after one-way ANOVA following Tukey’s multiple range test. Values are expressed as mean ± standard deviation (SD). ND: no data due to mortality.

The plasma inorganic components of *P. stellatus* after acute exposure to waterborne copper are shown in Figure 3. As with hematological analyses, plasma parameters were measured only in surviving individuals. Therefore, data presented in this section reflect physiological responses at copper concentrations from 0.5 to 12 mg Cu^2+^/L, as no fish survived at 24 and 48 mg Cu^2+^/L. Plasma calcium significantly increased at 12 mg Cu^2+^/L and above, while magnesium increased at concentrations of 0.5 mg Cu^2+^/L or higher (*p* < 0.05). Plasma organic components of the *P. stellatus* after acute exposure to waterborne copper are shown in Figure 4. Among plasma organic components, glucose and cholesterol levels significantly rose at 3 mg Cu^2+^/L and 0.5 mg Cu^2+^/L or higher, respectively, but total protein showed no significant changes (*p* < 0.05). The plasma enzyme components of *P. stellatus* after acute exposure to waterborne copper are shown in Figure 5. Plasma enzymes AST and ALT increased significantly at 3 mg Cu^2+^/L and above, whereas ALP levels remained unchanged across all concentrations (*p* < 0.05).

## 4. Discussion

Copper (Cu) is the most abundant transition metal found in nature, and it serves as an essential trace element for aquatic organisms at low concentrations. However, when aquatic organisms are exposed to copper levels exceeding a certain threshold, copper can accumulate in vital organs, disrupt physiological functions, and ultimately lead to mass mortality [7,24]. Oliveira et al. (2014) reported an LC_50_ value of 1.88 mg/L in fat snook (*Centropomus parallelus*, 3.5 ± 1.7 g, 6.8 ± 1.3 cm) after 96 h of acute waterborne copper exposure, attributing mortality to disruptions in Na^+^ and Ca^2+^ ion exchange in the gills [25]. Alkobaby et al. (2017) found an LC_50_ of 7.94 mg/L for Nile tilapia (*Oreochromis niloticus*, 2.97 ± 0.37 g, 5.39 ± 0.26 cm) under similar exposure conditions [26]. Oliva et al. (2009) reported a much lower LC_50_ of 0.32 mg/L in juvenile Senegal sole (*Solea senegalensis*, 2.08 cm, 0.07 g), suggesting mortality was due to irreversible histopathological damage affecting physiological health [27]. De Boeck et al. (2004) further demonstrated species-specific sensitivity by reporting 96 h LC_50_ values of 210 μg/L for rainbow trout (*Oncorhynchus mykiss*), 660 μg/L for common carp (*Cyprinus carpio*), and 1400 μg/L for gibel carp (*Carassius auratus gibelio*) [28]. These findings highlight how sensitivity to copper varies significantly across species and may also depend on factors such as size, age, and physiological condition. In the present study, the LC_50_ value for starry flounder (*Platichthys stellatus*) was determined to be 15.644 mg/L after 96 h of acute waterborne copper exposure. This relatively high value indicates that starry flounder exhibit a lower sensitivity to copper toxicity compared to the other fish species previously studied. The LC_50_ represents the concentration of copper at which 50% mortality occurs within a specific exposure period and serves as a key indicator of a species’ tolerance to toxic substances under defined environmental conditions. Given that copper toxicity and tolerance can be influenced by numerous factors—including species, developmental stage, body size, water temperature, salinity, and other environmental parameters—further studies under varying experimental conditions are essential to enhance the understanding of copper toxicity mechanisms and to support ecological risk assessments for diverse aquatic species.

Exposure to metals can alter hematological parameters in fish, potentially leading to increased erythrocyte hemolysis or, in severe cases, hematopoietic system damage resulting in hypoxia-induced mortality [19]. Fish blood is a key indicator of stress response to environmental changes, and hematological traits can vary depending on the type, concentration, and exposure duration of toxic substances [3]. Copper exposure is particularly associated with disruptions in iron metabolism, as iron is essential for heme synthesis. High copper levels can thus cause anemia and changes in blood indices [29]. Several studies have reported decreases in hemoglobin, RBC count, and hematocrit following copper exposure. For instance, Afaghi et al. (2020) observed reductions in hemoglobin and RBCs in *C*. *carpio* due to osmoregulatory dysfunction and erythrocyte damage [7]. Similar hematological declines were reported in catfish, *Clarias gariepinus* [30], streaked prochilod, *Prochilodus lineatus* [31], Catla, *Labeo catla* [32], and under chronic exposure in *C. carpio* [33]. In this study, *P. stellatus* also showed significant decreases in hemoglobin, RBC count, and hematocrit after acute waterborne copper exposure. This suggests that high copper concentrations disrupted the fish’s hematopoietic function, leading to impaired oxygen transport and blood cell damage. These findings indicate that copper toxicity had a significant physiological impact on *P. stellatus*.

Hematological indices such as mean corpuscular volume (MCV), mean corpuscular hemoglobin (MCH), and mean corpuscular hemoglobin concentration (MCHC) are useful indicators for assessing pollutant toxicity. HA et al. (2017) reported that chronic copper exposure reduced MCV in *O. niloticus* due to hemolytic anemia and reduced erythropoiesis [34], while Naz et al. (2023) found no significant change in MCH under similar conditions [35]. Al-Tamimi et al. (2015) observed a decrease in MCHC in Rohu, *Labeo rohita*, which was attributed to hemolysis or suppressed erythrocyte production [33]. In this study, *P. stellatus* exhibited significant reductions in MCV and MCHC after acute copper exposure, likely due to smaller and immature erythrocytes and reduced hemoglobin levels. MCH remained unchanged.

Plasma calcium and magnesium levels are key indicators of ion regulation and can reflect physiological stress from metal exposure [36]. Tavares-Dias et al. (2011), Baeck et al. (2014), Eyckmans et al. (2010), and Latif et al. (2014) all reported increases in these ions after copper exposure in various fish species, often linked to osmoregulatory dysfunction and gill or kidney damage [37,38,39,40]. In this study, *P. stellatus* also showed significant increases in plasma calcium and magnesium after waterborne copper exposure, suggesting impaired ion balance and osmoregulation.

Plasma glucose is widely used as a reliable indicator of physiological stress in fish, as its levels are sensitive to environmental disturbances such as heavy metal exposure [36]. Exposure to copper has been shown to increase glucose concentrations in several species. Monteiro et al. (2005) and Canli and Canli (2015) reported that chronic copper exposure in *O. niloticus* led to elevated glucose levels, which were attributed to disturbances in carbohydrate metabolism and stress-related hormonal responses [36,41]. Similarly, Heydarnejad et al. (2013) observed increased glucose in *O. mykiss* after copper exposure, suggesting that this was due to the breakdown of liver glycogen to meet the higher energy demand under stress conditions [42]. Atli et al. (2015) also found increased glucose levels following cadmium exposure in *O. niloticus*, indicating similar physiological responses under metal stress [43]. In this study, plasma glucose levels in *P. stellatus* significantly increased after acute exposure to waterborne copper. This result suggests that copper exposure triggered a stress response in the fish, leading to enhanced glycogenolysis to supply the energy needed to cope with the physiological imbalance.

Plasma cholesterol, a precursor of steroid hormones and a key membrane component, is often used as an indicator of metal-induced stress due to the damaging effects of metals on cell membranes [44]. Previous studies have reported increased cholesterol levels in fish following copper exposure. For instance, Mazandarani et al. (2017) observed elevated cholesterol in *C. carpio* after 14 days of subacute copper exposure, suggesting increased mobilization for cortisol synthesis under stress [29]. Similar increases were reported in *L. rohita* and bronze featherback, *Notopterus notopterus*, indicating copper-induced hypercholesterolemia [35,45]. In this study, plasma cholesterol levels in *P. stellatus* significantly increased after acute exposure to copper, suggesting a stress response associated with steroid hormone synthesis and membrane disruption.

Plasma total protein plays a key role in homeostasis and tissue repair and can serve as an energy source during toxic stress. Changes in total protein often reflect liver damage or protein loss, making it a useful indicator of metal toxicity like copper [42,46]. Some studies report a significant decrease in total protein after copper exposure in species such as Indian flying barb and Nile tilapia, depending on exposure duration and concentration [44,47]. However, other studies found no significant changes in total protein in fish like *O. niloticus*, *S. schlegelii*, and Caspian roach, *Rutilus caspicus* [36,48,49]. In this study, *P*. *stellatus* showed no significant change in total protein after acute copper exposure, suggesting limited effects on protein metabolism and liver function under these conditions.

Plasma enzymes AST and ALT are key in amino acid and protein breakdown and serve as important indicators of liver damage, as they are released into the blood when liver cells are injured [50,51]. Studies have shown increased AST and ALT levels after chronic copper exposure in species like *L. rohita* and *O. niloticus*, indicating liver damage caused by copper [35,44,52]. Similarly, increased AST and ALT were observed after manganese exposure in red seabream, *Pagrus major* and *S. schlegelii*, due to liver injury [53]. In this study, plasma AST and ALT levels rose following acute waterborne copper exposure, reflecting liver dysfunction and enzyme leakage. Alkaline phosphatase (ALP), an enzyme used to assess liver and kidney damage, showed no significant change after copper exposure here, suggesting limited effects on ALP activity under these conditions [53,54].

## 5. Conclusions

In conclusion, exposure to waterborne copper up to 12 mg Cu^2+^/L caused significant physiological and hematological changes in *Platichthys stellatus*, while higher concentrations (24 and 48 mg Cu^2+^/L) resulted in severe mortality, limiting further analysis. The 96 h LC_50_ was determined to be 15.644 mg/L, suggesting that *P. stellatus* is relatively less sensitive to copper toxicity compared to other fish species. Copper exposure led to anemia, disruptions in ion balance, stress responses, and liver impairment, as indicated by altered blood parameters and elevated liver enzymes. Toxic effects were evident even at low copper concentrations (1–3 mg/L). Although liver histological analysis was not conducted, it is recommended for future studies to better assess hepatotoxicity. These results emphasize the need to monitor copper contamination in aquatic environments to protect both ecosystems and human health, especially vulnerable populations such as children and pregnant women who are at higher risk due to fish consumption.

## Figures and Tables

**Table 1:** Lethal concentration (LC_50_) of starry flounder, *Platichthys stellatus*, exposed to waterborne copper for 96 h.

95% Confidence Limits	
Probability	Estimate (mg/L)
0.01	−3.107
0.10	5.315
0.20	8.861
0.30	11.418
0.40	13.602
0.50	15.644
0.60	17.687
0.70	19.871
0.80	22.428
0.90	25.974
0.99	34.396

## Data Availability

The data supporting the findings of this study are available from the corresponding author upon reasonable request.
